# Increasing cancer awareness and prevention in Africa

**DOI:** 10.3332/ecancer.2019.939

**Published:** 2019-07-25

**Authors:** Ahmedin Jemal, Otis W Brawley

**Affiliations:** 1 American Cancer Society, 250 Williams Street, Atlanta, GA 30303, USA; 2 Johns Hopkins University, 1550 Orleans Street, Suite 1M16, Baltimore, MD 21231, USA

**Keywords:** cancer control, cancer control implementation, cancer prevention, cancer awareness, Africa

## Abstract

Cancer awareness in the general population is an absolute essential and the basis on which a cancer-control programme can be constructed. Elements that go into cancer awareness and prevention efforts include knowledge of the problem and its solutions, a group of people who respect and care for the populations they want to serve, and resources. This paper discusses the importance of cancer awareness to cancer control and outlines some successful cancer awareness and control programmes in Africa. There is an audience in North America, Europe and Asia with resources that can be used. Awareness campaigns can also be used to recruit assistance and resources from governments, non-governmental organisations and pharmaceutical companies. Potential funders will provide support if they see a well-defined problem, a solution that is likely to be implemented and likely to work and organisations that can implement that solution.

## Background

The control of cancer is best brought about through a combination of cancer prevention and treatment [1]. Marketing, communications and social science are also important to increase cancer awareness. Population awareness of the cancer problem, seeing it as a priority and voicing achievable goals are all necessary elements of a successful cancer-control campaign.

Cancer prevention involves efforts to reduce the risk of cancer [2]. It is a long-term investment aimed at getting a large population to adopt healthy habits. It can be very effective and impact a large portion of a population. Cancer prevention is also general health promotion. Cancer prevention activities coincidentally lower risk of several other significant chronic diseases: cardiovascular disease, diabetes mellitus, orthopaedic joint degeneration and chronic obstructive pulmonary disease.

Cancer treatment requires significant infrastructure. The disease must be diagnosed and staged before therapy is ultimately administered. Therapy should include palliative and hospice care. Treatment is expensive, has limited effects and ultimately impacts a relative small portion of the population. Even resource-rich nations have difficulty providing adequate, high-quality treatment to all of their population in need [3].

## Cancer prevention

Compared to cancer treatment, cancer prevention is more efficient, more effective and less expensive. In the US, the majority of the more than 26% decline in the age-adjusted cancer mortality rate over the past quarter century is due to prevention, primarily tobacco-control efforts [4].

Much of prevention is health education. Ideally, one could maximise cancer prevention in a population by ensuring that everyone understood the most effective elements of cancer control. These messages must be based on science. Too often, well-meaning non-experts provide inaccurate and sometimes even harmful information. Basic, accepted cancer prevention information includes:

- Use of tobacco products is harmful and to be avoided.
- One should try to eat a balanced, calorie-restricted diet and exercise regularly.
- Several communicable diseases [HIV, HCV and Hepatitis B virus (HBV)] can cause cancer and other problems and one should work diligently to minimise the risk of contracting them.
- HBV vaccination can prevent liver cancer
- Human papillomavirus (HPV) vaccination of boys and girls can prevent cervical cancer and may prevent several other cancers.

Ideally, the population would practice healthy habits consistent with the above from an early age, carry them into adulthood and encourage them in their children. All people in a population being aware is ideal. Making a large proportion of a population aware is a more realistic goal.

## Health promotion messaging

We have established a general message of cancer health promotion and defined the ultimate audience. Let us discuss refining the message, the messenger and the conveyance of the message.

A specific population will have to deal with a number of challenges at any given time. That population best understands its challenges and best determines its priorities. A cancer awareness campaign can divert resources from more pressing issues. For this reason, message development must have substantial input from the population served. The message must be appropriate, culturally sensitive and tailored to the audience [5]. While the general health message can be developed with the help of outsiders, it must be refined by the members of the population who will use it—so too must the method of message conveyance be refined. As a general rule, cancer awareness messaging should convey why a problem is important and ask for an action to address the problem. Whenever possible, the intervention should be based on descriptive epidemiology—that is, data specific to the population addressed. The message should not exaggerate or sensationalise. It should not use fear-mongering to motivate action. At the same time, the message needs to be compelling. Awareness campaigns in the USA and Europe have often had difficulty avoiding the trap of exaggeration and fear mongering. When discovered, this can cause loss of trust among the population and undermine efforts.

While trying to bring about awareness in the general population, the campaign and its message also need to target societal leaders. The support of understanding and caring government officials (to include politicians and civil servants), officials of non-governmental organisations (NGOs) and community leaders at all levels is essential. Some of the most effective cancer prevention has been through governmental action [6]. These include: excise taxes on tobacco, restrictions on the sale and use of tobacco, and building codes that facilitate walking and exercise.

It is also desirable to make sure medical and educational leaders are aware and involved early on in an awareness effort. Healthcare providers must incorporate preventive activities and counselling in their medical practice. An important element of health promotion should be in the elementary school curriculum.

## Examples of awareness and prevention efforts in Africa

There are several examples of awareness and prevention efforts in Africa. Some are early and have led to announcements of planned improvements. Unfortunately, few of the older programmes had a formal planned assessment to fully determine results. The importance of a formal assessment is better appreciated in newer efforts.

As a health advocate, Princess Nikki of Nigeria has been very successful in inviting the spouses of African leaders to meet and discuss the breast and cervical cancer problem. The annual meeting includes 12 to 18 spouses of the heads of state of sub-Saharan African countries and public health experts from the USA, Europe and Africa. They discuss women’s health issues, defining the problem and potential solutions. This effort has brought cancer awareness to the highest reaches of several governments. A number of heads of state have designated staff to address cancer prevention and treatment issues. Plans have been announced to develop additional cancer hospitals in Kenya and to build four radiation therapy facilities in Ethiopia. Some governments have supported cervical screening and emphasised HPV vaccination.

Cervical cancer is a major cause of cancer death among African women. Visual inspection of the cervix with acetic acid (VIA) is a medical screening procedure aimed at finding and quickly treating cervical dysplasia. It has been shown to prevent cervical cancer and reduce the risk of death [7]. Dr Christine Kaseba, a Zambian first lady, who is a physician, used the Princess Nikki meetings to advocate for VIA. Cervical cancer awareness and education in Zambia is successfully conveyed by lay health educators who have been taught about cervical cancer issues and nurse midwives who have been taught to perform the procedure. Zambia has also established a network of village-based community health workers (equipped with mobile phones) to do health education. This word-of-mouth education is an example of respecting cultural sensitivities. This programme has been replicated in Rwanda, Mozambique, Namibia, Sudan and several other African countries [8]. Significant numbers of women have been screened over the past decade. Estimates of the number of abnormalities found and treated are only available for certain small programmes.

In 2009, Rwanda’s First Lady, Mrs Jeannette Kagame, met with Merck officials to discuss the availability of the HPV vaccine [9]. This led to a negotiation between Merck, the Rwandan Ministry of Health and members of Rwanda’s health sector. A public–private alliance of organisations that included GAVI, The Vaccine Alliance, UNICEF and the WHO joined in [10]. A national cervical cancer prevention strategy was developed. Using a school-based vaccination programme, they vaccinated more than 95% of the 98,800 Rwandan girls in sixth grade in 2012. A programme to vaccinate girls as they come of age continues [11]. Today more than a quarter of a million Rwandan girls have received full vaccination for HPV.

Several African countries, among them Zambia, Ethiopia, Kenya and South Africa, have a vital corps of health reporters. They use radio, television and print. Blogs and new media focused on health have appeared over the past decade. The web has not been exploited to its fullest potential. Special attention must be given to ensure that accurate information is provided in all media. The American Cancer Society, in collaboration with several African NGOs with a focus on cancer, has conducted cancer-control education sessions for the news media. This is helping reporters learn about the issues and how to write about them.

Tobacco companies are trying to expand into new markets as tobacco consumption declines in the USA and Europe [12]. Africa is ripe for exploiting. Increasing cigarette consumption is a major health problem in sub-Saharan Africa. Tobacco-control awareness efforts have blunted this rise using electronic and print media for broad dissemination of advice not to start smoking or to stop smoking if one already does. The health-promotion efforts of the news media have gone beyond tobacco control. They have focused on diet and exercise (energy balance), limiting alcohol consumption and responsible sexual activity.

The World Health Organisation Framework Convention on Tobacco Control (FCTC) is a United Nations treaty [13]. It was adopted by the World Health Organisation governing body in May 2003 and came into force in February 2005. The FCTC was signed and ratified by 180 of the 192 WHO member states. For these countries, the treaty is a multilateral and binding legal agreement setting standards for tobacco control. The standards regarding sale and packaging of tobacco will increase awareness of the harms of tobacco.

## Conclusion

Elements that go into cancer awareness and prevention efforts include knowledge of the problem and its solutions, a group of people who respect and care for the populations they want to serve, and resources. There is an audience in North America, Europe and Asia with resources that can be used. Awareness campaigns can also be used to recruit assistance and resources from governments, NGOs and pharmaceutical companies. In order to provide support, these entities have to see organisation, a well-defined problem and a solution that is likely to be implemented and likely to work.

## Conflicts of interest

The authors have no conflicts of interest to report.

## Funding statement

There was no specific funding provided for this article.
